# Primary Adenoid Cystic Carcinoma of the Upper Anterior Mediastinum Mimicking a Thyroid Tumor: A Case Report and Review of Literature

**DOI:** 10.3389/fendo.2020.00242

**Published:** 2020-04-23

**Authors:** Qiuji Wu, Weizi Sun, Jiajun Bu, Yuanhang Xiang, Yahua Zhong

**Affiliations:** ^1^Department of Radiation and Medical Oncology, Zhongnan Hospital of Wuhan University, Wuhan, China; ^2^Department of Oncology, Wuhan Fourth Hospital, Wuhan, China; ^3^Department of Pathology, Zhongnan Hospital of Wuhan University, Wuhan, China

**Keywords:** thyroid neoplasms, adenoid cystic carcinoma, diagnosis and treatment, case report, literature review

## Abstract

Primary adenoid cystic carcinoma (ACC) of the upper anterior mediastinum mimicking a thyroid tumor has rarely been seen in clinical practice and lacks a standard of care therapy. Here, we report a 47-year old female patient with an ACC originated from the upper anterior mediastinum presenting as a thyroid gland tumor. The patient received gross surgical resection of the tumor and underwent post-surgical chemotherapy and radiotherapy. The patient was free from local recurrence 3-years following initial treatment, but developed multiple lung metastases. She remains under clinical observation without discomfort and is still followed as an outpatient. Here, we also summarized recent reports of similar cases with hope to provide some experience for future clinical practice.

## Introduction

Adenoid cystic carcinoma (ACC) is one of the most common malignancy in salivary glands, accounting for 1–2% of all head and neck malignancies and about 10% of all salivary gland tumors (1). Although quite rarely seen, ACC may also be found in uncommon sites including the larynx and trachea, as well as the lung, where submucosal seromucinous glands are thought to give rises to these tumors (2). ACC deriving from the thyroid gland is extremely rare, with most cases reported originating from the upper respiratory tract and invading into the thyroid gland, mimicking a thyroid tumor (3, 4). Distinct cytological characteristics and immunohistochemical profiles are important basis for the differentiation of ACC from other classical thyroid tumors such as papillary adenocarcinoma and medullary carcinoma (5). Surgery remains the principal treatment for locally advanced ACC of the upper respiratory tract invading the thyroid gland. However, given the infiltrative properties and the perineuronal invasion activities of ACC, postoperative radiotherapy is often delivered with the purpose of improving local control (6). Distant metastasis of ACC may eventually form and pose a difficult clinical problem, as there are no efficient treatments for metastatic ACC.

The most common sites of metastasis include the lung (20%), the bones (4%), and the liver (3%) (7). Three large retrospective studies using public database comprising more than 2,000 patients showed 5-year OS from 78 to 90.3%, 10-year OS from 59.6 to 79,9%, while 15-year OS differed from 35 to 69.2% (8–10). The mostly indicated prognostic factors included pathological types, patient age, disease stage, surgical margin status, site of origin, perineural invasion, etc. (11, 12).

Comprehensive understanding of molecular mechanisms of ACC might bring new opportunities to improve the prognosis of this disease. Critical molecular alterations such as *MYB-NFIB* translocation, *Sox 4, c-KIT*, and *Slug* gene activation, overexpression of epidermal growth factors (EGFs), vascular endothelial growth factors (VEGFs), nerve growth factors (NGFs), and their corresponding receptors have been demonstrated to play essential roles in the pathogenesis of ACC by recent studies (13). New targeted therapies such as anti-EGFR or anti-VEGF monoclonal antibodies provide potential anti-tumor activities, but larger clinical studies are warranted to evaluate their clinical benefits (14). A better understanding of the molecular mechanisms underlying ACC pathogenesis and tumor development is therefore in urgent need for novel and more efficient anti-tumor strategies.

Thyroid invasive tracheal ACC is a rare clinical presentation. Here, we report a 47-year old female patient with a thyroid gland tumor that was confirmed by post-surgical pathology to be an ACC originated from the upper anterior mediastinum, most likely from the trachea. We describe the clinical manifestations, diagnosis, treatment, and follow-up of this patient, with a hope to provide some experience for future clinical practice.

## Case Presentation

**Figure 1:**
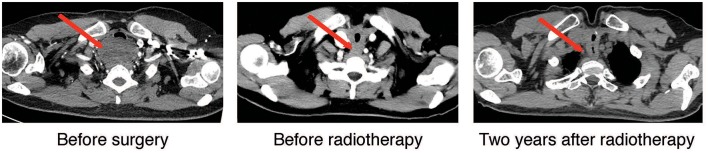
Cervical-thoracic CT scans of the patient. Representative axial images of the disease before surgery, post-surgery/before radiotherapy, and 2-years after radiotherapy were shown. The red arrow highlighted the tumor and post-treatment changes in the tumor area.

**Figure 2:**
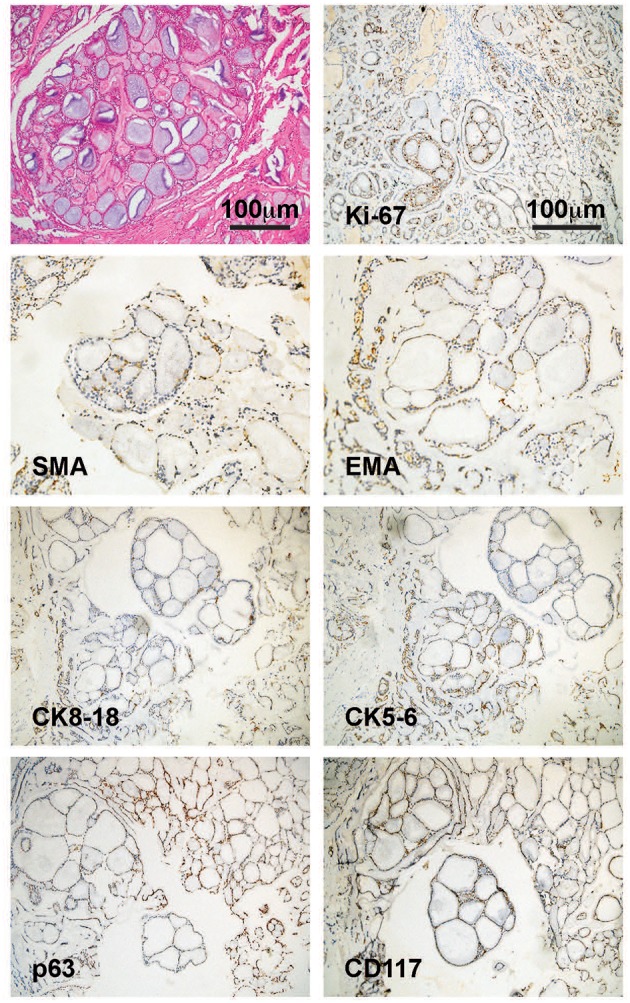
Pathological features of the tumor. HE staining image indicated a cribriform ACC. Immunohistochemical staining images showed positive expression of Ki-67, SMA, EMA, CK8-18, CK5-6, p63, and CD117, which supported a diagnosis of ACC. ACC, adenoid cystic carcinoma; SMA, smooth muscle actin; EMA, epithelial membrane antigen; CK, cytokeratin; CD, cluster of differentiation.

A 47-year old female with no significant past medical history presented with a thyroid mass and a foreign body feeling when swallowing. The patient denied palpitation, dysphoria, fever, insomnia, cough, dyspnea, breathlessness, hoarseness, or other symptoms. The Doppler ultrasonography showed a hypoechoic solid mass about 67 × 40 mm with blood flow signals posterior to the lower border of the right lobe of the thyroid. Another hypoechoic solid mass about 70 × 43 mm with blood flow signals posterior to the lower part of the left lobe of the thyroid gland was also noted. Multiple enlarged lymph nodes with the largest ones measuring 18 × 6 mm in the right neck, and 15 × 5 mm in the left neck, were also detected. She then received a computed tomography (CT) scan of the neck and thorax that revealed a 85 × 54 mm hypodense-to-isodense space-occupying lesion in the posterior superior mediastinum, one solid mass in the right lobe, and another solid mass in the inferior posterior part of the left lobe of the thyroid. The lesions showed slight enhancement with a contrast-enhancement scan and caused compression of the trachea, esophagus and cervical vessels (Figure 1, left panel). An esophagogastroscopy also revealed stenosis of the esophagus 15–20 cm distal to the upper incisors. Two months following the initial presentation, the patient underwent surgical treatment with right and left thyroidectomy, upper mediastinal tumor resection, left recurrent laryngeal nerve exploration, right recurrent laryngeal nerve anastomosis, and tracheotomy. During the surgery, the thyroid gland was found to be adherent to the surrounding tissue and a tumor measuring about 10 × 8 × 6 cm was present within the dorsal part of the right lobe of the thyroid gland was noted. Specifically, the tumor was between the trachea and esophagus, but the border of the tumor was indistinct. The tumor involved the tracheal membranous wall, the upper esophagus, and the right recurrent laryngeal nerve. The left lobe of the thyroid gland was not directly involved by the tumor. Pathological investigations demonstrated an ACC of the upper mediastinum involving the left lobe of the thyroid gland, the tracheal cartilage, and the adjacent muscles. The immunohistochemical staining profile was as follows: Alcian blue (AB) (+), Periodic Acid Schiff (PAS) (–), CD56 (–), Syn (–), Calponin (+), smooth muscle actin (SMA) (+), P63 (+), epithelial membrane antigen (EMA) (+), cytokeratin (CK)5/6 (+), CK8/18 (+), CD117 (+), thyroid transcription factor-1 (TTF-1) (–), thyroglobulin (Tg) (–), chromogranin A (CgA) (–) (Figure 2). Perineuronal invasion was also noticed. The mass in the left lobe of the thyroid gland turned out to be a nodular goiter.

**Figure 3:**
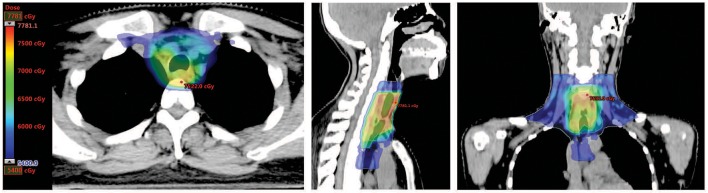
Dose distribution of post-surgical radiotherapy. Representative axial, sagittal, and coronal images of dose distribution were shown. Areas with dose coverage of over 54 Gy were presented.

Postoperative CT scan showed changes in the area of the thyroid, with thickening of the upper thoracic esophageal wall, but no obvious abnormalities elsewhere in chest (Figure 1, middle panel). Other examinations including abdominal CT and bone scintigraphy were negative. The thyroid profile was as follows: free T3 3.27 pmol/L, free T4 10.76 pmol/L, TSH 48.400 μIU/mL, anti-Tg 12 IU/mL, anti-TPO 6 IU/mL. The patient was supplemented with oral thyroid hormone. The patient then received one cycle of chemotherapy in the form of 75 mg/m^2^ of Cisplatin and 75 mg/m^2^ of Docetaxel but were canceled because of intolerable gastrointestinal side effects. Since the tumor involved the tracheal cartilage and showed perineuronal invasion, radiation therapy was proposed. The patient underwent postoperative intensity-modulated radiation therapy (IMRT) with a prescribed dose of PTV-GTV of 70 Gy, PTV-CTV1 of 60 Gy, PTV-CTV2 of 54 Gy in 31 fractions (1 fraction per day, 5 fractions per week; Figure 3). She experienced grade II leucopenia, but the treatment was otherwise well tolerated.

The patient returned for post-treatment follow-up visit 3 months after the radiation therapy. CT scan showed no obvious changes compared with previous scans. The patient has undergone scheduled follow-up with CT scans as routine imaging since then.

Local control was quite satisfactory and the patient was free of local recurrence (Figure 1, right panel). However, multiple tiny nodules appeared in both lungs 2-years later following the initial presentation showing very slow increase in size. However, she declined any further treatment. The patient is still under follow-up 3-years after initial treatment and is not reporting any discomfort.

## Discussion

ACC mainly occurs in salivary glands with an relentless clinical course. However, local recurrence and distant metastasis are frequent in ACC patients due to the infiltrative characteristics of the tumor (15). Although ACC might also occur in other sites such as the trachea and the lung, primary thyroid ACC has not been described. To date, there were only few reported cases of ACC arising from the adjacent tissue of the thyroid such as the larynx and the trachea, where it invades into the thyroid gland mimicking a primary tumor. While other even rarer cases of thyroid metastasis of ACC from distant organs such as the salivary glands were also reported (Table 1).

**Table 1:** Cases of ACC with thyroid invasion reported in literature.

**References**	**Sex**	**Age**	**Origin location**	**Size (cm)**	**Symptoms**	**Other metastasis location**	**Management**	**Survival (Months)**
Kukwa et al. (4)	F	17	Trachea	3.7 × 2.6	Wheezing	Bilateral lungs	RR + RT (70 Gy)	>12 (live)
Aldrees et al. (16)	F	47	Trachea	3 × 4	Neck swelling, cough, shortness of breath, and hoarseness	None	RR	NA (live)
Nuwal et al. (17)	F	44	Trachea	2.5 × 2	Neck swelling	NA	RT (50 Gy)	Lost to follow-up
Shirian et al. (18)	M	45	Larynx	NA	Mild hoarseness and left-sided neck mass	NA	RR	NA
Qi et al. (3)	M	46	Trachea	NA	Dysphagia and associated dyspnea	NA	Bilateral partial mass resections	1 (dead)
Kashiwagi et al. (19)	F	33	Larynx	NA	Cough and wheeze	NA	RR	>24 (live)
Wang (20)	M	57	Trachea	NA	Cough, pressure and suffocation in the chest	Bilateral lungs	CH, RT (60 + 60 Gy), Apatinib	>120 (live)

*F, female; M, male; NA, not available; RR, radical resection; RT, radiation therapy; CH, chemotherapy; ACC, adenoid cystic carcinoma*.

Cytologically, ACC is characterized by monomorphic basaloid small round or oval tumor cells with round nuclei, small indistinct nucleoli, scant cytoplasm, and no inclusions, ranging in strands or clumps surrounding globules of acellular, homogenous spaces filled with mucoid or hyaline material (17). Three pathological patterns of ACC have been well-described, namely the cribriform, trabecular and solid types, with the cribriform type being the most prevalent. Cribriform ACC shows distinct microscopic “Swiss cheese” morphology and contains mucinous material strongly stained by alcian blue. Immunohistochemically, smooth muscle actin, S100, and vimentin, which are absent in thyroid malignancies. Primary thyroid tumor typically expresses thyroglobulin (Tg), thyroid transcription factor-1 (TTF-1), while these markers are absent in ACC (17).

Although CT scan and fine needle aspiration cytology (FNAC) are important diagnostic tools for thyroid tumors (17). ACC is rarely seemed in the thyroid gland and therefore misdiagnoses may occur. Post-surgical thorough histopathological examination and immunohistochemical procedures are therefore essential for an accurate diagnosis and proper differential diagnosis (5).

Surgery remains the major treatment for locally advanced ACC. However, the infiltrative properties of ACC render most surgeries non-radical. In our case, the tumor not only invaded the right lobe of the thyroid gland, but also involved the upper anterior mediastinum and the tracheal cartilage and displayed perineuronal infiltration. Therefore, postoperative radiotherapy was given in order to ensure optimal local control. Indeed, after 3-years of surgery and radiotherapy, the local control was satisfactory. However, lung metastases have developed.

On the other hand, the outcome of systemic treatment in metastatic ACC is far from satisfactory. In general, ACC is not sensitive to a variety of chemotherapeutic agents, although drugs including Cisplatin, Fluorouracil, and Taxol have shown low to moderate response rate (21). Novel targeted therapies such as anti-EGFR such as Cetuximab, and the small molecule inhibitor of VEGFR2, Apatinib, have entered clinical trials and yielded potential clinical benefit in recurrent or metastatic ACC patients (20, 22). Other agents targeting distinct molecular alterations such as *c-Kit* overexpression, *MYB-NFIB* fusion, *MYB* overexpression, and NF-κB over activation are still under investigations and might provide more choice for ACC patients (23).

Since ACC represents an indolent disease and patient with lung metastasis could still have relatively long overall survival (median OS 32.3 months), re-surgery for lung oligometastasis or close follow-up and best supportive care for multiple non-resected lung metastasis might also be a choice (23). For instance, Kukwa reported a case of 17-year old female patient who developed lung metastasis 3-years after the treatment of primary tracheal ACC with thyroid metastasis (4). The patient underwent metastasis resection and no recurrence was detected 1-year after the surgery.

In conclusion, although ACC is less sensitive to radiotherapy than squamous cell carcinoma, it is still important treatment to reduce local recurrence. The long-term prognosis of remains ACC is still dismal, with the 10 and 20-year OS being 52 and 28%, respectively (24). The main cause of death is distant metastasis, in particular lung metastasis (24). Further research is needed to improve the survival of ACC patients. For patients with ACC invading the thyroid gland who underwent thyroid gland removal and post-operative radiotherapy, thyroid hormone therapy is also critical.

## Data Availability Statement

All datasets generated for this study are included in the article.

## Ethics Statement

The studies involving human participants were reviewed and approved by Medical Ethics Committee of Zhongnan Hospital of Wuhan University. The patients/participants provided their written informed consent to participate in this study. Written informed consent was obtained from the individual(s) for the publication of any potentially identifiable images or data included in this article.

## Author Contributions

QW and WS designed the study and wrote the manuscript. JB and YX analyzed pathological and imaging data and participated in discussion. YZ supervised the study and corrected the manuscript.

## Conflict of Interest

The authors declare that the research was conducted in the absence of any commercial or financial relationships that could be construed as a potential conflict of interest.
